# Genome-Wide Scans for Ghanaian *Plasmodium falciparum* Genes Under Selection From Local and Chinese Host Populations

**DOI:** 10.3389/fcimb.2021.630797

**Published:** 2021-02-25

**Authors:** Shan-Mei Shi, Tian-Qi Shi, Shen-Bo Chen, Yan-Bing Cui, Kokouvi Kassegne, Moses Okpeku, Jun-Hu Chen, Hai-Mo Shen

**Affiliations:** ^1^ National Institute of Parasitic Diseases, Chinese Center for Disease Control and Prevention, Key Laboratory of Parasite and Vector Biology of the Chinese Ministry of Health, National Centre for International Research on Tropical Diseases, WHO Collaborating Center for Tropical Diseases, Shanghai, China; ^2^ School of Global Health, Chinese Centre for Tropical Diseases Research, Shanghai Jiao Tong University School of Medicine, Shanghai, China; ^3^ Discipline of Genetics, School of Life Science, University of Kwazulu-Natal, Durban, South Africa; ^4^ National Institute of Parasitic Diseases, Chinese Centre for Disease Control and Prevention⁃Shenzhen Centre for Disease Control and Prevention Joint Laboratory for Imported Tropical Disease Control, Shanghai, China

**Keywords:** *Plasmodium falciparum*, Ghana, imported malaria, variant surface antigen, positive selection, acquired immunity

## Abstract

Initial malarial infection mostly causes symptomatic illness in humans. Infection that is not fatal induces complete protection from severe illness and death, and thus complete protection from severe illness or death is granted with sufficient exposure. However, malaria parasite immunity necessitates constant exposure. Therefore, it is important to evaluate lowered immunity and recurrent susceptibility to symptomatic disease in lower transmission areas. We aimed to investigate selection pressure based on transmission levels, antimalarial drug use, and environmental factors. We whole genome sequenced (WGS) *P. falciparum* clinical samples from Chinese hosts working in Ghana and compared the results with the WGS data of isolates from native Ghanaians downloaded from pf3k. The *P. falciparum* samples were generally clustered according to their geographic origin, and Chinese imported samples showed a clear African origin with a slightly different distribution from the native Ghanaian samples. Moreover, samples collected from two host populations showed evidence of differences in the intensity of selection. Compared with native Ghanaian samples, the China-imported isolates exhibited a higher proportion of monoclonal infections, and many genes associated with RBC invasion and immune evasion were found to be under less selection pressure. There was no significant difference in the selection of drug-resistance genes due to a similar artemisinin-based combination therapy medication profile. Local selection of malarial parasites is considered to be a result of differences in the host immunity or disparity in the transmission opportunities of the host. In China, most *P. falciparum* infections were imported from Africa, and under these circumstances, distinct local selective pressures may be caused by varying acquired immunity and transmission intensity. This study revealed the impact of host switching on the immune system, and it may provide a better understanding of the mechanisms that enable clinical immunity to malaria.

## Introduction

Malarial parasites have yielded to the selection pressure of host immune systems after coexisting and interacting with their hosts for over 150 million years. (Carter and Mendis, 2002). It is well known that the immune system of a human host can block parasite development at different stages; but, malarial parasites have long been evolving in response to threats from multiple immune mechanisms (Rénia and Goh, 2016). It is expected that new immune escape mechanisms will constantly be uncovered. Although not always, initial malaria infection tends to cause symptomatic illness in humans. (Ryg-Cornejo et al., 2016). In this process, infants may develop lethal febrile illness, but adults acquire complete protection from severe illness or death, even though some believe that sterile immunity can never be achieved (Simon et al., 2015).

Over the last decade, the number of malaria cases globally has reduced by at least half, and many of the malaria endemic countries around the world will navigate gradual elimination with predictable results (Ariey et al., 2019). Similar to other pathogens, parasite immunity is acquired through constant exposure. Therefore, research has focused on lowered immunity and recurrent susceptibility to symptomatic disease in lower transmission areas (Barry and Hansen, 2016). During this long-term fight between humans and malaria, almost all surface antigens of the parasite have been tested for vaccine development, and their extensive polymorphisms from geographical distribution are considered to slow down the development of acquired immunity (Doolan et al., 2009; Barry and Arnott, 2014).

Researchers noted that selection pressure on parasites varies with location due to several reasons, including varying transmission ecology, innate susceptibility of mosquitoes or human hosts, degrees of acquired immunity in humans, and drug pressure in certain areas (Crompton et al., 2014). Duffy et al. emphasized that in highly endemic areas, malaria faces genotype competition due to superinfections, with stronger host acquired immune responses (Duffy et al., 2015). More genome-wide analyses of *P. falciparum* confirmed this conclusion and revealed a significant global population structure (Manske et al., 2012; Zhu et al., 2019).

After decades of efforts to control this illness, China has reduced its malaria burden from 2,961/100,000 population in 1970 to 0 in 2017, and aims to eliminate malaria nationwide (Feng et al., 2018). Today, the burden weighs mostly in Sub-Saharan Africa, where the largest prevalence rates are found (Mbacham et al., 2019; Papaioannou et al., 2019). Researchers reported 8,653 P. falciparum imported cases from 2011 to 2015. These cases were imported from 41 sub-Saharan countries into China, leading to 98 deaths, mostly of Chinese laborers (Lai et al., 2016).

To test whether differences in selection emerge due to different hosts, patterns relating to both transmission level and drug use and environmental variations, Ghanaian *P. falciparum* samples from two different human host populations were analyzed. Clinical isolates from Chinese patients who either worked or have worked in Ghana were whole genome sequenced (WGS) and compared with the WGS data from native Ghana residents downloaded from pf3k (https://www.malariagen.net/projects/pf3k). Our clinical isolates were collected during a large-scale outbreak of imported malaria in 2013 (Li et al., 2015). Isolated parasites resembled those from the Ghanaian population. Our comparative analysis also revealed extensive genetic diversity, different selection signatures, and host adaptation-focused genomic plasticity.

In this study, we scanned the difference in *P. falciparum* genes under selection pressure to evaluate the impact of declining transmission on clinical immunity. Samples collected from two host populations showed evidence of differences in selection intensity. The imported population exhibited a higher proportion of monoclonal infections, and many genes associated with RBC invasion and immune evasion were found to be under low selection pressure. Our results deepen our understanding of factors that might drive clinical immunity against malaria.

## Materials and Methods

### Sampling *P. falciparum* Parasites and Genome Sequencing

A malaria outbreak comprising 874 patients was reported in Shanglin County, China in 2013, and 871 of these patients were confirmed to be overseas laborers who had returned from Ghana. Blood samples were collected from these imported cases; all samples tested microscopically positive, and a PCR confirmed single *P. falciparum* infection. However, after rapid on-site disposal and treatment, only nine samples with high parasite density were retained for follow-up sequencing. DNA was extracted from blood samples using the QIAGEN DNeasy Blood & Tissue Kit (Qiagen, UK), and sheared into 500 bp fragments to construct the libraries with Covaris S2 (Covaris, Inc., USA) instrument. All libraries on Illumina X-10 were sequenced and generated an average of 109M (34–540 M) paired-end reads of 150 bp. All Illumina raw sequencing reads were submitted to the Chinese National Sharing Service Platform for Parasite Resources. In addition, we downloaded the *P. falciparum* 3D7 reference sequence from the PlasmoDB database (Aurrecoechea et al., 2008).

All sequenced raw reads were filtered by removing the adapter and low-quality sequences with Trimmomatic-3.0 (Bolger et al., 2014) and mapped to the 3D7 reference sequence using Burrows-Wheeler Aligner (Li and Durbin, 2010). Genotyping was performed using in-house R script based on GATK4 best-practice workflows and recalibrated with pf3k standard known-site files (Mckenna et al., 2010). High-quality single nucleotide polymorphism (SNP) data were derived by excluding SNPs with >5% missing calls in each sample; missing calls were defined as positions with <2 reads. We downloaded high-quality SNP data from 92 indigenous Ghana *P. falciparum* isolates collected in 2013 from the PF3k project to build the reference data set (Amenga-Etego, 2012; Mensah-Brown et al., 2015).

### Population Structure, Genetics Analysis, and Selection Tests

We assessed the population structure using both, the global collection and our imported isolates. Principal component analysis (PCA, Person n-1) was performed using the R4.02 package. Analysis of the ancestry shared between individual isolates was performed using the ADMIXTURE package (Alexander and Lange, 2011) to check the relationship between the China-imported and native Ghanaian isolates and their respective populations. In these analyses, the SNP dataset was filtered to exclude each locus with a minor allele frequency (MAF) of <5% for all positions, which had been restricted to loci that appeared at least once in both our samples and the references.

In this study, we used the within-isolate *F*_WS_ fixation index to determine the within-infection genomic diversity in relation to the total population using the bahlolab/moimix R package (Lee, 2015). Isolates with an *F*_WS_ score of 1 were considered as a single predominant genotype. Two of the samples in our study had genetically mixed infections (*F*_WS_ *<*0.95), analogous to inbreeding coefficient. We then checked the heterozygosity calls to evaluate genotypic errors. For two or more segregating allele loci across the two samples, we discarded the minority calls (the allele with low read depth) and retained the major allele only. Without hypnozoite-induced relapses, genetic complexity was considered to not likely affect our genetic analysis process.

In the population genetics section, we followed the evolution analysis pipeline from an earlier study, in which genome-wide variation was undertaken on clinical isolates from highly endemic regions of Guinea and compared with those of Gambia (Mobegi et al., 2014). We estimated the nucleotide diversity $\left(\hat{\pi }\right)$, Watterson’s estimator $\left(\hat{\theta }\;\omega \right)$, genetic differentiation (*F*_ST_), and Tajima’s D value across all of the genes in ARLEQUIN-Ver3.5 (Excoffier and Lischer, 2010) on the SNP dataset of both populations.

Meanwhile, we used the integrated haplotype score (iHS) test to calculate the standardized log ratio of integrated extended-haplotype homozygosity on nine imported samples in Selscan-Ver1.10a (Szpiech and Hernandez, 2014). The XP-EHH test was also performed using the same software to obtain the standardized log ratio of the integrated site-specific EHH between the China-imported and the native Ghanaian populations.

## Results

### Genomic Data Summary

Variant call format (VCF) files of 92 indigenous Ghanaian *P. falciparum* isolates collected in 2013 were downloaded from the PF3k project (www.malariagen.net/pf3k) to build the reference data set. After the quality control process was completed, a total of nine samples with high-quality genomic data was included in the analysis. The samples generated paired-end reads with an average read length of 150 bp. A variable proportion of reads from the nine isolate samples were mapped to the *P. falciparum* 3D7 reference. An average of 95% convergence in the whole genome was defined as high-quality consensus base calls. Using the GATK Genotype, we uncovered a total of 109,173 SNP loci, of which <5% contained missing calls (**Table 1**).

**Table 1:** Sequencing and mapping summary statistics for nine China imported samples.

Sample ID	Total Reads	Mapped ratio (%)	Average coverage depth (X)	Genome covrage >1x (%)	Covrage >10x (%)	F_WS_
Ah_28	56,712,364	16.25	51.09	99.16	92.47	0.7400
D_Pf4_1	81,054,920	7.42	29.57	98.10	81.62	0.9594
PFDL_15	49,232,000	4.97	5.73	95.05	9.46	0.9724
PFDL_17	50,636,206	5.49	8.27	97.69	27.00	0.9552
PFGX_121	47,107,990	6.48	9.86	98.84	41.81	0.8587
PFGX_154	55,644,848	9.35	20.63	98.78	86.74	0.9587
PFGX_212	47,977,732	4.46	4.29	90.18	5.06	0.9692
PFGX_316	58,322,570	2.44	6.69	96.97	18.04	0.9766
PFGX_81	56,278,848	5.46	8.62	97.32	30.34	0.9598

### Complexity of Infection

We checked the within-sample parasite diversity of the imported samples to determine the proportion of single-genotype isolates. An *F*_WS_ value of >0.95 indicates that an infection predominantly contains a single genotype; thus, a higher proportion of imported samples were monoclonal infections. The distribution of *F*_WS_ scores was similar across isolates, ranging from 0.73 to 0.97 (mean 0.92). Seven isolates had *F*_WS_ values above 0.95, indicating that the samples were centered on single genotypes. Within each isolate, the majority allele at each SNP was included in the population-based allele frequency analysis.

### Population Structure of *P. falciparum* Isolates

**Figure 1:**
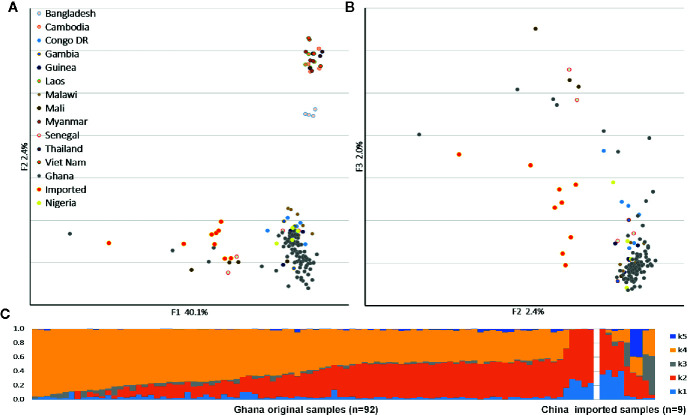
Parasite population structure in nine China-imported samples relative to the reference. **(A)** Principal component analysis (PCA) plots illustrating the genetic differentiation between populations around the world. **(B)** Within Ghanaian population, samples collected from different hosts were divided along the secondary axis. **(C)** ADMIXTURE bar plot illustrates the population structure within Ghanaian populations from local and Chinese hosts at an optimized cluster value of K = 5.

We applied PCA to the SNPs to compare the population structures with reference samples from around the world (**Figures 1A, B**). The *P. falciparum* samples clustered generally according to their geographic origin, and the samples collected from different hosts were divided along the major axis. The imported cases showed a clear African origin, and a similar, yet by no means identical, distribution with the native Ghanaian samples. The ADMIXTURE analysis identified several parts that correspond to each component of the samples (**Figure 1C**). In accordance with the general assumption, the imported samples appeared as a mixture of all the components and demonstrated the full range of the Ghana isolates, but the native Ghanaian samples comprised only K2, K4, and a few K1 components. Components K3 and K5 were almost absent in native samples, implying a higher degree of acquired immunity in African hosts.

### Genomic Scan for Differentiation Between Populations

**Figure 2:**
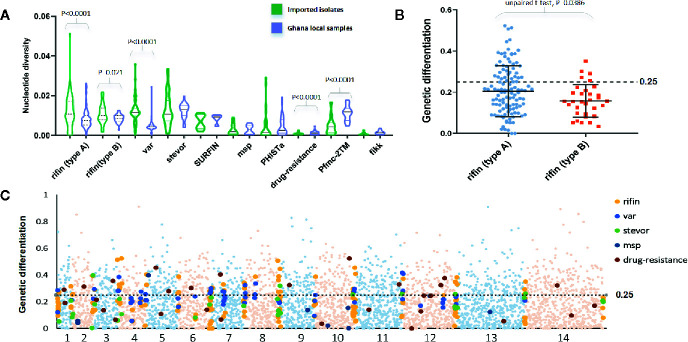
*F*_ST_ value of all genes summarizing the difference between China-imported samples (n=9) and native Ghanaian (n=92) reference samples. **(A)** The variation profile of some gene families exhibited greater genetic diversity between imported and local samples. **(B)** *F*_ST_ value of each gene from two subgroups of the *rif* family showed significant differences. **(C)** *F*_ST_ value in all genes for pairs of populations to explore genomic effects of local and overseas hosts.

In a total of 5,602 genes, we estimated the average $\hat{\pi }$ and $\hat{\theta }\omega$ values to b 0.0013 and 0.0016, respectively. As expected, the mean values were significant lower than the native Ghanaian samples (P<0.0001, one-tailed z-test); the $\hat{\pi }$ and $\hat{\theta }\omega$ values were estimated to be 0.0017 and 0.0035, respectively. The variation profile of some gene families, particularly those associated with RBC invasion and immunity, exhibited greater genetic diversity than all gene backgrounds (**Figure 2A**), such as *rifin* (0.0116) and *var* (0.012). Based on a putative drug-resistance gene list from previous studies (Gamo et al., 2010; Plouffe et al., 2016; Cowell et al., 2018), we checked the diversity of these genes from Chinese and Ghanaian hosts. For 37 of the most common drug-resistance genes (**Supplementary Table 1**), the average $\hat{\pi }$ value was 0.0007 in imported samples and 0.0013 in native samples.

We calculated the *F*_ST_ value in individual genes for pairs of populations to explore the genomic effects from local and overseas hosts (**Figure 2C**). Although these two populations should be considered to have the same Ghanaian lineage, they still exhibited considerable differences for each gene (mean *F*_ST_ = 0.13, median = 0.33); 1252 genes had *F*_ST_ values >0.25. Among the high *F*_ST_ value genes, those associated with the organism membrane (GO:0044279 and GO:0044218), protein binding (GO:0005515), and important processes, such as stimulus response (GO:0050896) and interspecies interaction (GO:0044419), were found to be significantly enriched. For the variant surface antigen (VSA) genes, only a small part (54 *rif*, 7 *stevor*, but no *var*) appeared in the high *F*_ST_ value list, which is in accord with the fact that receptor adhesion phenotypes of RBCs normally depend on a few specific variant surface antigens that are expressed. For example, two subgroups of the *rif* family showed significant (P = 0.038, *t*-test) differences in the *F*_ST_ test (**Figure 2B**). RIFINs mediate the resetting of pRBCs and demonstrate stronger geographic differences, whereas B-RIFINs showed lower variability in molecules and expression. Meanwhile, 13 drug-resistance genes such like *mdr1*, *apiap2*, and *crt* showed higher *F*_ST_ values, reflecting the differences in treatment policy between the two countries (Ehrlich et al., 2020).

**Figure 3:**
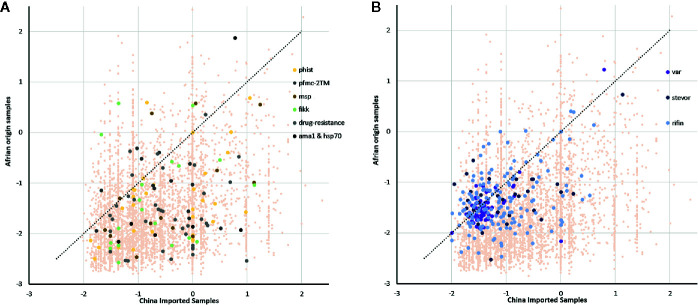
Tajima’s D value of all genes summarizing the difference of balanced selection between China-imported samples (n=9) and native Ghanaian (n=92) reference samples. The lower mean value in local samples suggested a stronger selection pressure from African hosts. **(A)** Some important genes/gene families showed the same trend as that of the gene background, and these genes/gene families include phist, 2TM, msp1, and drug-resistance genes. **(B)** Unlike the background, there was not much difference in variant surface antigen (VSA) gene families between the imported and local groups.

We identified signatures of selection pressure in individual genes to reveal divergence between different host populations (**Supplementary Table 2**). Not surprisingly, the two Tajima’s D values were mostly negative, with an average of -0.66 and -1.58 in imported and local samples, respectively (median = -0.85 and -1.76), and mean value of imported samples were significantly higher than local samples (P <0.0001, z-test for paired samples). The background of all genes was consistent with earlier studies that described a historical population expansion of *P. falciparum* in Africa (Loy et al., 2017), and the lower mean value in native Ghanaian samples also suggested stronger selection pressure from African hosts. Similar to the gene background, the mean Tajima’s D value was less negative in drug-resistant genes from the imported group (mean =-0.45, median =-1.17) compared with that from the native group (mean =-1.6, median =-1.44) (**Figure 3A**), which also occurred in some other infection-related gene families, such as *2TM* (mean =-0.57 and -1.05, median =-0.61 and -1.04), *fikk* (mean =-0.68 and -1.31, median =-0.93 and -1.52), and *msp1* (mean = − -0.39 and − -1.18, median = − -0.58 and − -1.69) (Bachmann et al., 2015). However, not much difference within VSA gene families was detected (**Figure 3B**) (average for *rif*, *var*, and *stevor* were -1.06, -1.35, and -1.01 in the imported group and -1.35, -1.47, and -1.27 in the native group, respectively).

### Scan for Evidence of Directional Selection

**Figure 4:**
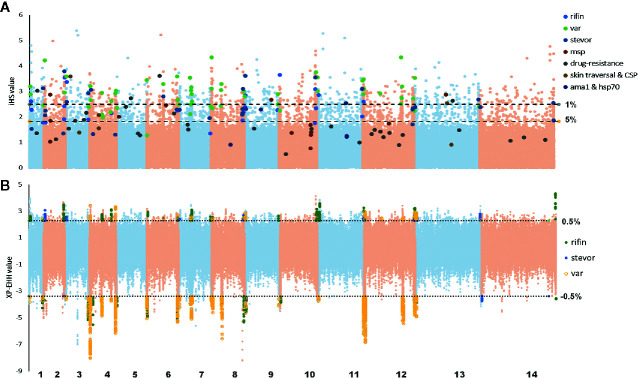
Haplotype-based detected positive selection in China-imported samples. **(A)** Genome-wide scan of standardized |iHS| for single nucleotide polymorphisms (SNPs) with a minor allele frequency (MAF) of at least 5% in imported samples. Dashed lines indicate the top 1% and 5% of |iHS| values (|iHS| score >2.49 and 1.82, respectively). SNPs from important gene or gene families with top values were listed. **(B)** Genome-wide scan of standardized XP-EHH for SNPs with a MAF of at least 5% in China-imported samples, using native Ghanaian samples as the reference population. Dashed lines indicate the top 1% of XP-EHH values (± 0.5%, XP-EHH score >2.29 or <-3.39 in imported/local populations). SNPs from the VAS gene family with top ranking values were listed.

In this study, we used the iHS statistic to detect incomplete sweeps and identified SNPs above the top 1% value of the randomly expected distribution at the whole-genome level (**Table 2** and **Supplementary Table 3**). Usually, in the *Plasmodium* genome, the top selected SNP locus is associated with RBC invasion and immune evasion genes. In our imported population, we found that the top 1% SNPs involved 329 genes, including 46 *var*, 5 *msp*, 66 *rif*, and 12 *stevor* (**Figure 4A**). In addition, most of these gene family members are present in the top 5% list (59 *var*, 130 *rif*, and 26 *stevor* in 788 genes), and these genes are located close to each other on the chromosome. This condition is common in malarial parasites because positive selection could increase the prevalence of both, the selected variant as well as of nearby variants, and local regions of extended haplotypes were generated. For the drug-resistance genes, only four genes, *mcp1*, *pi3k*, *ap2tf*, and *K13*, showed higher iHS values (**Table 2** and **Supplementary Table 1**) and reflected the unrestricted use of artemisinin-based combination therapies (ACTs) in African countries (Gallien et al., 2013; Tun et al., 2015; Velasco et al., 2017).

**Table 2:** Notable *P. falciparum* genes with their associated positive selection statistics.

Chromosome	Gene	Product	$\hat{\pi }$	Tajima’s D test	FST	Top |ihs| value	Top XP-EHH value
**Mosquito transmission**							
13	PF3D7_1335900	thrombospondin-related	0.012	0.472	0.244	2.542	
**Liver Stage Infection**							
3	PF3D7_0304600	Circumsporozoite protein	0.005	0.222	0.350	3.595	
**Erythrocytic stage evasion**							
2	PF3D7_0206800	merozoite surface protein 2	0.009	-1.335	0.150	2.881	
4	PF3D7_0421300	PfEMP1	0.013	-1.397	0.401	2.627	2.436
6	PF3D7_0617600	stevor	0.009	-0.947	0.115	2.806	2.461
7	PF3D7_0711700	PfEMP1	0.013	-1.374	0.534	3.550	2.460
9	PF3D7_0930300	merozoite surface protein 1	0.005	0.060	0.312	2.676	
9	PF3D7_0937500	rifin	0.015	-1.314	0.326	2.509	2.653
9	PF3D7_0900100	PfEMP1	0.010	-1.668	0.220	2.810	2.903
11	PF3D7_1149900	stevor	0.025	-1.160	0.655	3.109	2.483
12	PF3D7_1255100	rifin	0.010	-0.878	0.400	2.982	3.234
12	PF3D7_1255000	rifin	0.013	-0.941	0.373	2.529	3.221
13	PF3D7_1300200	rifin	0.010	-1.841	0.396	2.803	2.785
**Drug-resistance**							
1	PF3D7_0108400	mitochondrial carrier protein	0.001	-1.478	0.116	3.0285	
5	PF3D7_0515300	phosphatidylinositol 3-kinase	0.001	-1.074	0.674	2.732	
6	PF3D7_0613800	AP2 domain TF	0.002	-0.546	0.110	3.613	-3.701
13	PF3D7_1343700	kelch protein K13	0.000	-0.552	0.368	2.642	

XP-EHH was also applied to compare the average haplotype length associated with each SNP between the imported and native Ghanaian references. The selection signals were obviously stronger in native Ghanaian samples and concentrated in VSA genes (68 VSA in 96 genes) (**Table 2** and **Supplementary Table 4**). In imported isolates, in addition to VSA, these stronger selection signals were also located in some gene families, such as *pfmc-2TM* and *surfin*. However, unlike the local samples, these top signals of selection encompassed multiple genes, rendering it difficult to focus on specific genes (**Figure 4B**).

## Discussion

Malarial parasites have already coexisted with their hosts for over 150 million years. Throughout their life cycle, these parasites exhibit great immune evasion ability, even if they are at the asymptomatic liver stage, by using sophisticated system of proteins to avoid immune recognition (WHO, 2016) in the blood stage. Various immune evasion strategies comprise a major obstacle to the development of effective therapeutics. In as early as the 1950s, MacGregor et al. found that antibodies were a key component of antimalarial immunity (Preston et al., 2014). Decades later, researchers have characterized many immune targets and potential vaccine candidates and identified antigen-specific immune responses associated with malaria infection protection. Many studies have focused on biomolecular, genetic, and immune strategies in both hosts and parasites to better understand protective anti-*Plasmodium* immunity, but there is still a long way to go (WHO, 2016).

To date, malaria is one of the major causes of death in the African continent; the African continent accounts for about four-fifths of all cases of the world in 2017 alone, with 4% of these cases occurring in Ghana (Xu and Liu, 2016). Meanwhile, half a world away, the malaria burden in China has been dramatically reduced from 2961/100,000 in 1970 to 0.1/100,000 in 2014, and eventually down to 0 in 2017 (Zhou et al., 2016). The elimination progress soon hit a barrier since now imported malaria cases have been reported in almost all provinces in China. In 2013, a malaria outbreak (874 cases in total) was reported in the Guangxi province of China, most occurring in miners returned from Ghana (Wang et al., 2017). In response, Shanglin County conducted mass malaria screening and found an attack rate of 216/1,000 that was much higher than expected.

In the last 10 years, the number of malaria cases has fallen sharply worldwide. Malarial immunity is acquired through constant exposure, and in lower transmission areas, more studies have focused on declining immunity in communities and the recurrent susceptibility to symptomatic disease. For example, Fowkes et al. reported that rebounds of malarial infections occurred in low transmission areas (Chen et al., 2018). For similar reasons, the lack of acquired immunity in Chinese citizens increased their susceptibility to *P. falciparum*. Thus, we scanned the differences in genes under selection pressure between Chinese and Ghanaian hosts to reveal the impact of declining transmission on clinical immunity. We expected the samples to cluster according to their geographic origin and exhibit more detail in the population structure. We also hypothesized that the imported population should exhibit a higher proportion of monoclonal infections, and many genes/gene families associated with RBC invasion and immune evasion are under less selection pressures. In the native Ghanaian population, we suspected the samples to encounter a strong acquired immunity response from local human hosts and cause strong positive selection. Therefore, we expected to observe signals of extended haplotype homozygosity on immune evasion related genes. We also estimated that there would be no significant difference in the selection of drug-resistance genes due to the same ACT-based medication profile in both countries.

Our results are consistent with these assumptions. In the global population structure, samples were clustered according to their geographic origin, and imported isolates, which could be regarded as part of the Ghanaian population, maintained slight diversity to the reference sequence. It was quite unexpected that overall genetic diversity was lower in imported samples (the mean $\hat{\pi }$ values in the imported and native samples were 0.0013 and 0.0017, respectively); even though we assumed low selection pressure from Chinese hosts would enable parasites to mutate faster. However, when it comes to genes associated with RBC invasion and immunity, greater diversity was exhibited in imported samples than local ones, such as *rif* (0.0116), *var* (0.012), and *stevor* (0.013). The VSA gene families exhibited the greatest diversity in the parasite, and our observations were consistent with previous studies (Volkman et al., 2007). Highly polymorphic proteins have been used to mediate antigenic variation and help malaria parasites escape immune clearance (Julien and Wardemann, 2019). We found a higher proportion of monoclonal infections, and the average *F*_WS_ scores were higher than those in a local study during the same period (mean values of 0.81 and 0.74 in the Kintampo and Navrongo provinces of Ghana, respectively) (Duffy et al., 2015). A high ratio of monoclonal infection could be due to a low entomological inoculation rate or clonal parasite population. Chinese laborers normally worked in small enclosed spaces, with less chance of superinfection or co-transmission.

As we delved into evolutionary analysis, we could see natural selection acting at the gene level. The host immune response along with its genetic background is essential for malaria prevention. Clinical immunity to malaria is definitely acquired, even though the process might be slow in areas of stable transmission. The significantly higher average Tajima’s D values (*P <*0.0001, *z*-test for paired samples) of imported samples demonstrated a lack of acquired immunity in Chinese citizens, while 3,934 genes showed negative values. However, in local samples, almost all genes were under directional selection (5,253 genes with negative values). This difference revealed that continued exposure to malarial antigens maintains immunity. For example, sporozoites use microneme (SPECT-1, PLP1, and TRAP) and some circumsporozoite (CSP) proteins to overcome skin and hepatic immune defenses during their invasion (Lo et al., 2016; Ryg-Cornejo et al., 2016; WHO, 2016). In these related genes, we detected stronger selective pressure in native Ghanaian samples (mean D value -0.002 in imported and -0.36 in local group), consistent with the background.

Meanwhile, in the erythrocyte stage, the key evasion mechanism is sequestration, which is mediated by proteins, such as PfEMP-1, RIFIN, and STEVOR that allow iRBC adherence to the vascular endothelium, thereby avoiding clearance (Langhorne et al., 2008; Wahlgren et al., 2017) and sequestering the cells in the microvasculature of various organs. As part of the immune evasion system, the VSA family did not encounter stronger selection, and the difference in Tajima’s D test on VSA gene families was quite narrow. While the XP-EHH test showed stronger selection in both populations concentrated in the VSA family, these signatures of selection were scattered in different individual genes, such as *crt* (PF3D7_0709000), in the local host group and imported group. Therefore, our results indicate that, with their tremendous genetic diversity, the VSA families launched different genes for particular hosts to ensure antigenic variation, maintain stable function, and demonstrate regional characteristics.

## Conclusions

Local selection of malaria parasites is considered to be due to differences in host immunity or disparities in transmission opportunities of the host. In China, most *P. falciparum* infections were imported from Africa, and under these circumstances, distinct local selective pressures may be caused by varying acquired immunity and transmission intensity levels. Against the background of similar structures, samples collected from two host populations showed evidence of differences in selection intensity. Compared with native Ghanaian samples, the China-imported population exhibited a higher proportion of monoclonal infections, and many genes/gene families associated with RBC invasion and immune evasion were under low-level selection pressures. There was no significant difference in the selection of drug-resistance genes due to a similar ACTs-based therapy. Our results deepen our understanding of factors that might drive clinical immunity against malaria.

## Data Availability Statement

All data supporting these findings are contained within the manuscript and supplementary tables. All Illumina raw sequencing reads were submitted to the Chinese National Sharing Service Platform for Parasite Resources (URL: https://www.tdrc.org.cn/portal-tdrc/a/freeLoginmatmain/matmain/form?id=207872). Sequencing data are available from the corresponding author upon reasonable request.

## Ethics Statement

The study was conducted based on the principles expressed in the Declaration of Helsinki. Following the study protocol, potential risks and benefits were explicitly explained to participants. Blood collection was performed with written informed consent of the participants and following institutional ethical guidelines that were reviewed and approved by the ethics committee at the National Institute of Parasitic Diseases, Chinese Center for Disease Control and Prevention.

## Author Contributions

J-HC and H-MS conceived and designed the experiments. S-MS, T-QS, S-BC, and Y-BC conducted the experiments. S-MS, T-QS, and H-MS analyzed the data. KK contributed reagents and materials. MO contributed to the analysis tool. S-MS and H-MS drafted the manuscript. MO and J-HC revised the manuscript critically in regard to intellectual content. All authors contributed to the article and approved the submitted version.

## Funding

This work was supported by the National Research and Development Plan of China (Grant No. 2018YFE0121600) and the National Sharing Service Platform for Parasite Resources (Grant No. TDRC-2019-194-30), the Project of Shanghai Science and Technology Commission (Grant No. 18490741100), the Foundation of National Science and Technology Major Program (Grant no. 2018ZX10734–404, 2016ZX10004222–004, and 2012ZX10004-220), and the National Natural Science Foundation of China (81101266). The funding bodies had no role in the design of the study, collection, analysis, and interpretation of data, or in writing of the manuscript.

## Conflict of Interest

The authors declare that the research was conducted in the absence of any commercial or financial relationships that could be construed as a potential conflict of interest.

## Supplementary Material

Supplementary Table 1 37 drug-resistance/putative genes summary with |iHS| and XP-EHH value.

Supplementary Table 2 5,602 *P. falciparum* genes with population genetic statistics (nucleotide diversity, Watterson’s estimator, Tajima’s D test, and *F_ST_ test*).

Supplementary Table 3 Genes with top 1% in integrated haplotype score.

Supplementary Table 4 Genes with top 1% (±0.5%) cross population extended haplotype homozygosity value in imported/local populations.

## Abbreviations

ACTs, artemisinin-based combination therapies; iHS, integrated haplotype score; PCA, Principal component analysis; PfEMP1, *P. falciparum* erythrocyte membrane protein 1; RBCs, red blood cells; RIFINs, repetitive interspersed families of polypeptides; STEVOR, subtelomeric variant open reading frame; VSAs, variant surface antigens; XP-EHH, cross-population extended haplotype homozygosity.
